# Synthesis of N-Doped Micropore Carbon Quantum Dots with High Quantum Yield and Dual-Wavelength Photoluminescence Emission from Biomass for Cellular Imaging

**DOI:** 10.3390/nano9040495

**Published:** 2019-04-01

**Authors:** Xin Ren, Fang Zhang, Bingpeng Guo, Na Gao, Xiaoling Zhang

**Affiliations:** 1 School of Chemistry and Chemical Engineering, Beijing Institute of Technology, Beijing 100081, China; shoushidarenxin@163.com (X.R.); wwlldd@163.com (B.G.); 15877319357@163.com (N.G.); 2 Analytical and Testing Center, Beijing Institute of Technology, Beijing 100081, China; fzhang2006@163.com

**Keywords:** carbon quantum dots, high quantum yield, multi-color PL emissions, imaging agents, photochemistry

## Abstract

Pursuit of a simple, fast, and cost-effective method to prepare highly and dual-wavelength fluorescent carbon quantum dots (CQDs) is a persistent objective in recent years. Here, we fabricated N-doped micropore carbon quantum dots (NM-CQDs) with a high quantum yield and dual-wavelength photoluminescence (PL) emission from sustainable biomass using a pulsed laser ablation method. Interestingly, two coexisting indigo–blue photoluminescence (PL) emissions were clearly observed, elucidating that the excited electrons transited from the intrinsic π* orbital to the surface state (SS) formed from the saturation passivation. The quantum yield (QY) and fluorescence lifetime (FL) of the obtained NM-CQDs were as high as 32.4% and 6.56 ns. Further investigations indicated that the emission behaviors of NM-CQDs were still stable and independent in various conditions such as various excitation wavelengths, salt ionic concentrations, pH values, irradiation times, and temperatures. The obtained NM-CQDs are very suitable for cellular staining images due to strong and stable PL emission and show good internalization in different cells. Therefore, we propose a new and cost-effective preparation strategy for highly fluorescent NM-CQDs with great potential in biomedical imaging and engineering.

## 1. Introduction

Carbon quantum dots (CQDs) are a promising material, featuring fluorescent carbon nanoparticles of less than 10 nm in size with a high surface-to-volume ratio [1,2,3]. Compared with other semiconductor QDs materials, such as CdS QDs, PbS QDs, etc., CQDs offer many advantages including good biocompatibility [4], robust chemical inertness [5], low toxicity [6], and outstanding optical properties [7], making them a promising carbon-based nanomaterial for applications such as biological sensing [8,9], bioimaging and photodynamic therapy [10,11,12,13], drug delivery [14] and analysis [15], light-emitting diodes (LEDs) [16], and solar cells [17].

To date, CQDs were synthesized via various methods which can be generally classified into two categories, “bottom-up” approaches and “top-down” approaches. In the “bottom-up” approaches, organic [18,19,20] or natural substances are carbonized [21] to fabricate CQDs, and “top-down” approaches feature cutting large-sized carbon materials [1,22,23,24,25] (e.g., graphite, activated carbon, carbon nanotubes, graphene oxide, carbon soot, etc.) into small-sized CQDs. Among the various approaches, the acidic exfoliation method, hydrothermal method, and microwave-assisted treatment method are the most common and dominant approaches. Generally, these methods are relatively complex, expensive, and environmentally hazardous in preparation processes of CQDs. Most of the obtained CQDs have only one photoluminescence (PL) emission with size-dependent and surface-passivation-dependent properties, which limit their further applications in dual-wavelength fluorescent emissions. In addition, some impurities are always produced due to the introduction of a strong acid in the preparation process of CQDs, resulting in the PL mechanism of CQDs being ambiguous. Therefore, it is very important to understand the PL mechanism of CQDs, especially for CQDs with dual-wavelength PL emissions.

Compared with the abovementioned methods, pulsed laser ablation (PLA) is a unique and novel approach, which is simple, cost-effective, and environmentally friendly in the fabrication process of carbon-based nanomaterials without any impurities [26]. Anomalous reactions and growth of the fragmented species can occur under non-equilibrium conditions, such as high temperature and high pressure in liquid. Many efforts showed the possibility for the synthesis of carbonic nanomaterial by nanosecond- or femtosecond-laser ablation of highly oriented pyrolytic graphite (HOPG) [27,28,29]. At present, the study of CQDs is in a new era. However, there are still many challenging issues to be solved, including the following: (1) carbon precursors for the preparation of CQDs by pulsed laser ablation are high-cost and non-renewable [28,30]; (2) the fluorescence quantum yield of CQDs is relatively low in many solvents such as water and ethyl acetate oleamide (often less than 20%) using the pulsed laser ablation method [27,28], which limits their applications in biomedical imaging and optoelectronics; (3) the PL mechanism of CQDs, especially for dual-wavelength PL mechanism, remains ambiguous. Therefore, it is necessary to propose a new and cost-effective preparation strategy to obtain pure CQDs with a high quantum yield and dual-wavelength PL emission for an understanding of the PL mechanism and application in biomedical imaging and engineering.

In this work, N-doped micropore CQDs (NM-CQDs) with a high quantum yield and dual-wavelength PL emission were fabricated using pulsed laser ablation from low-cost and sustainable *Platanus* biomass rather than using expensive carbon precursors. Unlike most reports, the NM-CQDs passivated by formamide solvent presented two coexisting and excitation-independent PL emission peaks, elucidating that the excited electrons transited from the intrinsic π* orbital to the surface state (SS). The PL intensities of NM-CQDs were stable after nine days, and the PL quantum yield and average fluorescence lifetime (FL) were 32.4% and 6.56 ns, respectively. Further investigations indicated that the PL emission behaviors of the formamide-passivated NM-CQDs were still stable in various conditions. In addition, the obtained NM-CQDs are very suitable for cell bioimaging and show good internalization in different cells. Therefore, we propose a new preparation strategy for highly fluorescent NM-CQDs with great potential in biomedical imaging and engineering.

## 2. Materials and Methods

### 2.1. Preparation of Highly Fluorescent NM-CQDs

**Figure 1:**
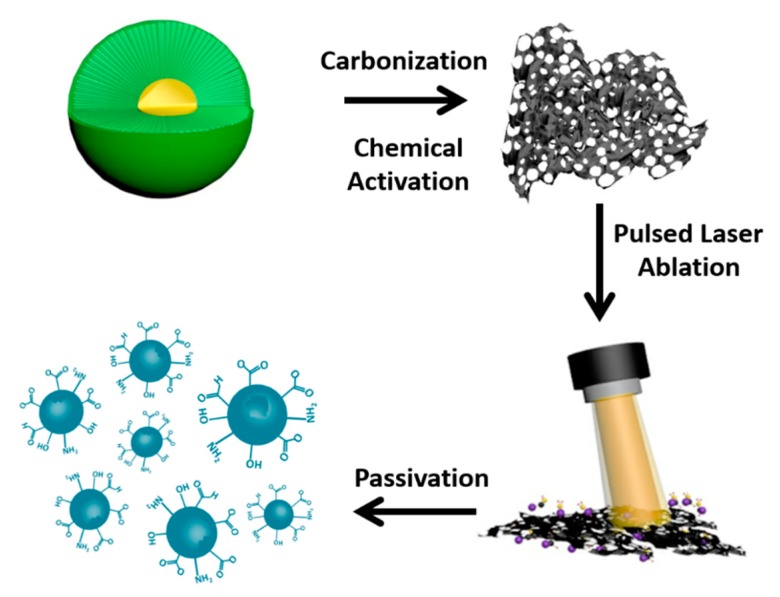
Synthesis process scheme of the N-doped micropore carbon quantum dots (NM-CQDs) derived from waste *Platanus* biomass.

Figure 1 shows the synthesis process scheme of NM-CQDs derived from waste *Platanus* biomass. Firstly, fruits of waste *Platanus* biomass were washed clean with deionized (DI) water, and were then dried in a vacuum-drying cabinet. Subsequently, the dried fruits were carbonized in a tube furnace filled with N_2_ at 600 °C for 2 h. Then, 0.5 g of carbonized sample was mixed with 1 g of KOH in 20 mL of DI water. The mixture was sonicated and agitated for 30 min, and then dried in a vacuum cabinet again. Finally, the mixture was chemically activated in a tube furnace to form a microporous carbon material at 900 °C. For investigating the effect of micropores, another comparative experiment was performed without using KOH. Briefly, 0.5 g of carbonized powder was directly mixed with 20 mL of DI water, and then the mixture was sonicated and agitated for 30 min. The sample was dried and further carbonized to form a non-microporous carbon precursor in a tube furnace at 900 °C.

The porous carbon (PC) powder was dispersed in 5 mL of formamide solvent in a cuvette, and the opening was covered with a polystyrene cap. The cuvette was placed on a three-dimensional (3D) stage. The mixture solution was ablated by a pulsed laser beam with a continuously adjusting 3D stage. After that, the solution was centrifuged at 10,000 rpm five times. Finally, the yellow solution containing NM-CQDs was obtained. Here, apart from formamide, ethyl acetate and ethylene glycol solvent were also used in the preparation of CQDs, and they were called M-CQDs-EA and M-CQDs-EG, respectively. In addition, N-CQDs were prepared by pulsed laser ablation of the non-micropore carbon precursor target in formamide. Broadly speaking, the wavelength and the repetition rate of the neodymium-doped yttrium aluminum garnet (Nd:YAG) pulsed laser were 1064 nm and 10 Hz, respectively. The laser energy and the pulse width were modulated to around 20 mJ and 3–6 ns. The ablation time was about 30 min; the homemade pulsed laser ablation system is shown in Figure S1 (Supplementary Materials).

### 2.2. Characterization of Highly Fluorescent NM-CQDs

The surface morphology of the PC was imaged on a field-emission scanning electron microscope (Model: JSM-7500 F, JEOL Ltd., Tokyo, Japan). TEM and high-resolution TEM (HR-TEM) images of NM-CQDs were taken on a transmission electron microscope (Model: JEM 2100, JEOL Ltd., Tokyo, Japan). Specific areas and porosities of sample were acquired on an automated gas sorption analyzer (Model: BELSORP-MAX, Bel Japan Inc., Osaka, Japan). The atomic force microscopy (AFM) image of NM-CQDs was captured on an atomic force microscope (Model: Park System XE-100, Park Systems Corp., Sungnam). Measurements of X-ray photoelectron spectra (XPS) were performed on an ESCA lab 250 spectrometer. The X-ray powder diffraction pattern was acquired on a Brooker Model D8 superspeed with a scan speed of 10°/min. Fourier-transform infrared spectra (FTIR) were recorded with a Nicolet IS10 spectrophotometer. Raman spectra were recorded with a Renishaw spectrophotometer with an excitation wavelength of 532 nm. The zeta potential measurements and dynamic light scattering (DLS) measurements were performed on a Nano ZS90. Measurements of absorption spectra were performed on an ultraviolet–visible light (UV–Vis) spectrophotometer (Model: TU-1901, Beijing Purkinje General Instrument Co., Ltd., Beijing, China). Fluorescence spectra and absolute quantum yield (QY) were measured by a fluorescence spectrometer (Model: Edinburgh FLS 920 and Edinburgh FLS 980, Edinburgh Instruments Ltd., Livingston, England). Time-resolved photoluminescence was recorded on an Edinburgh spectrometer (Model: F900) with a 375-nm laser as an excitation source. Fluorescence images were recorded on a confocal laser scanning microscope (Model: Olympus-FV1000, Olympus Corp., Tokyo, Japan).

### 2.3. Bioimaging of NM-CQDs in Different Types of Cells

Three different types of cells (HeLa cells, L02 cells, and macrophage cells) were employed for conducting cell imaging of NM-CQDs. Firstly, the different cells were incubated in 96-well dishes in a humidified incubator under 5% CO_2_ atmosphere for 24 h. Then, 40 µL of the NM-CQDs was introduced to different wells filled with HeLa cells, L02 cells, and macrophage cells, and the wells were further incubated at 37 °C for 3 h. Afterward, the culture medium was removed, and cells were washed with phosphate-buffered saline (PBS) solution (0.01 M, pH = 7.4) three times. Finally, the images of cells stained with NM-CQDs were obtained on a confocal laser scanning microscope. The excitation wavelength of the laser source was 405 nm.

## 3. Results and Discussion

**Figure 2:**
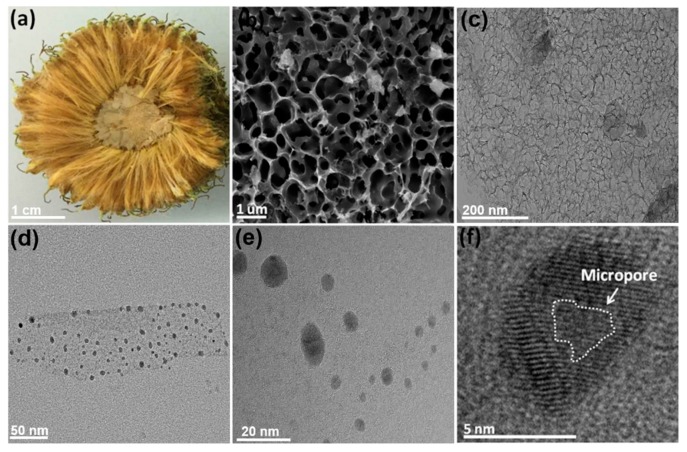
(**a**) Photograph of *Platanus* biomass. (**b**,**c**) SEM and TEM images of porous carbon (PC) after KOH activation. (**d**–**f**) TEM and high-resolution TEM (HR-TEM) images of NM-CQDs.

Figure 2 shows the topography images of NM-CQDs derived from waste *Platanus* biomass. Notably, the fruit of *Platanus* biomass is yellow in color and in a loose flocculating form (as shown in Figure 2a). The mixture of carbonized *Platanus* biomass and KOH was activated to prepare the porous carbon nanomaterial at 900 °C (as shown in Figure 2b). The corresponding TEM image is given in Figure 2c, which indicates that the nanocarbon precursor had an abundantly pored structure after activation. Meanwhile, the N_2_ sorption isotherm of pored carbon was of a type-I sorption characteristic (see Figure S2a, Supplementary Materials) [30]. Furthermore, the specific surface area was as high as 1690 m^2^/g. In addition, the pore size distribution ranged from 0.9 nm to 1.8 nm (as shown in Figure S2b, Supplementary Materials), which was assigned to the microporous structure. These results indicate that the pored carbon precursor is very suitable for preparing high-QY CQDs with great potential in biomedical imaging because more surface defects from the pored structure can induce more electron transition pathways, further facilitating the PL emission [31,32]. TEM images of the as-prepared NM-CQDs by PLA are shown in Figure 2d,e. Obviously, these NM-CQDs were monodispersed. Statistically, the average size of the as-prepared NM-CQDs by PLA was 8 nm, and most of them were less than 10 nm in size, which is in accordance with the Gaussian fitting (see Figure S3a, Supplementary Materials). The result is also consistent with the dynamic light scattering (DLS) spectrum of NM-CQDs (see Figure S3b, Supplementary Materials). The HR-TEM image of NM-CQDs in Figure 2f reveals a lattice fringe of 0.23 nm, which was ascribed to the (101) plane of layered graphite crystal [33]. Also, there was a micropore (approximately 1.4 nm) in the center of NM-CQDs. As shown in Figure S4a (Supplementary Materials), the AFM morphology of NM-CQDs indicates that heights of the obtained particles were 1.19, 1.23, and 1.66 nm along the black line. The majority of them were approximately 1.26 nm high (see Figure S5b, Supplementary Materials), which confirms that NM-CQDs possess a structure with a few layers after PLA. Herein, these results show that the NM-CQDs were fabricated from low-cost, sustainable, waste *Platanus* biomass by PLA for the first time.

**Figure 3:**
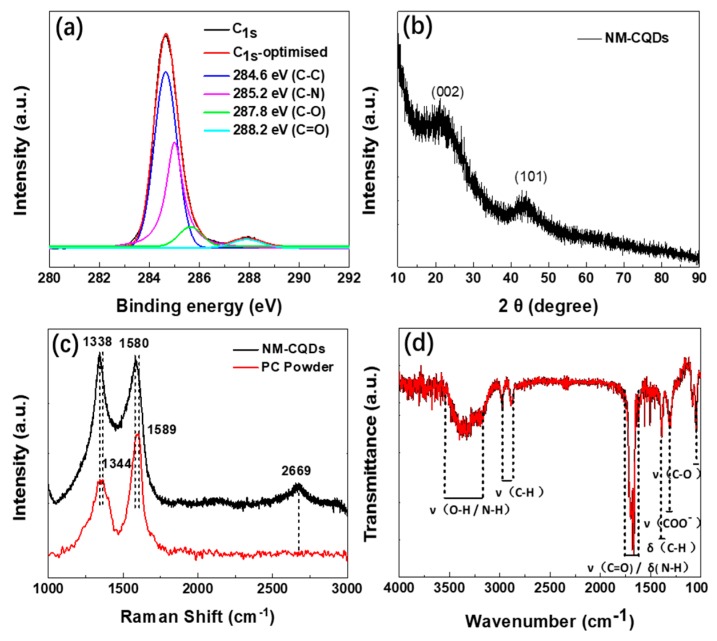
(**a**) High-resolution C_1*s*_ spectrum of NM-CQDs (red curve), and four fitting components (blue, magenta, green, and cyan curves). (**b**) X-ray diffraction (XRD) patterns of NM-CQDs. (**c**) Raman spectra of NM-CQDs and PC powder with an excitation wavelength of 532 nm. (**d**) Fourier-transform infrared (FTIR) spectrum of NM-CQDs.

Firstly, we performed XPS measurements to confirm the chemical constituents of the as-prepared NM-CQDs [34]. XPS analysis revealed that the three distinct strong peaks were mainly C (77%), N (10%), and O (13%) (see Figure S5a, Supplementary Materials), which were located at 285 eV (C_1*s*_ peak), 399 eV (N_1*s*_ peak), and 532 eV (O_1*s*_ peak), respectively. The corresponding high-resolution energy spectrum of the NM-CQDs in Figure 3a was assigned to four bands, which were C=O at 288.2 eV, C–O at 287.8 eV, C–N at 285.2 eV, and C–C at 284.6 eV. The binding energy peak located at 284.6 eV revealed the *sp*^2^ graphitic structures [19], and the peaks of C=O and C–O demonstrated carboxyl or other functional groups on the NM-CQDs. In order to further analyze the structure of the NM-CQDs, the N_1*s*_ high-resolution XPS was fitted by the Lorentzian method, as shown in Figure S5b (Supplementary Materials). Clearly, there is an intense band centered at 399.3 eV, which was attributed to the O=C–NH bond [34,35], indicating that surface passivation groups on the NM-CQDs existed in the form of amides. As shown in Figure 2b, the XRD pattern shows that NM-CQDs had two diffraction peaks. The (002) peak at 2θ = 23.2° was assigned to an amorphous carbon phase [28], and the latter (101) peak at 2θ = 44.3° was attributed to a lattice parameter with the interplanar spacing of 0.23 nm in TEM [5]. In addition, Raman spectroscopy, the most convincing fingerprint, was used to analyze the structure and layers of NM-CQDs. The three characteristic Raman peaks are shown in Figure 3c, indicating that NM-CQDs still possessed a unique hexagonal layered structure as a PC nanomaterial. The peak located at 1580 cm^−1^ was attributed to the G band, and the other two peaks located at 1338 cm^−1^ and 2669 cm^−1^ were attributed to the D band and 2D band, respectively [36]. Compared with that of bulk porous carbon precursor, the D and G peaks of NM-CQDs were mainly red-shifted by 6 cm^−1^ and 9 cm^−1^, respectively, which was attributed to the change of interlayer interaction of the as-prepared sample after pulsed laser ablation of the PC solution [37]. This phenomenon was similar to the cases of MoS_2_ and black phosphorus (BP) quantum dots [38,39]. Furthermore, the relative intensity of the “defect” D peak to the crystalline G peak was 1.02, which was higher than that of bulk PC (0.599), indicating that NM-CQDs had more defects than bulk PC. In addition, presence of the 2D peak of NM-CQDs indicates that the obtained samples possessed a structure with a few layers [36]. Finally, the surface structure of NM-CQDs was further confirmed by FTIR (see Figure 3d). The strong peak at 1658 cm^−1^ could be attributed to overlapped C=O and N–H bonds. The broad band located from 3556 cm^−1^ to 3174 cm^−1^ was attributed to the stretching vibrations of O–H bonds or N–H bonds. The stretching vibrations of C–H bonds were found at 2982 cm^−1^, 2874 cm^−1^, and 1398 cm^−1^. The peaks at 1308 cm^−1^ and 1054 cm^−1^ could be attributed to the stretching vibrations of COO^−^ bonds (zeta potential ~13.5 mV in Figure S6, Supplementary Materials) and C–O bonds, respectively [34,40,41]. The presence of C=O and N–H bonds, as surface passivation groups, indicated that carboxyl or amino moieties existed on the NM-CQDs, in accordance with the XPS measurement. During the PLA process of fabricating NM-CQDs in formamide, bulk porous carbon powder rapidly absorbed the localized high energy within a micro area, and was cracked into porous *sp*^2^ nanoclusters with a few layers, i.e., the skeleton of NM-CQDs. Meanwhile, formamide solvent molecules were also pyrolyzed by a laser with sufficient energy to form oxygen-containing and nitrogen-containing groups (e.g., carboxyl and amino moieties, etc.). The cracked functional groups rapidly passivated C atoms on the surface and edges of *sp*^2^ nanoclusters with a few layers to form the electron-donating substituents of NM-CQDs. Finally, the CQDs that consisted of porous *sp*^2^ nanosized domains with a few layers and electron-donating substituents on the periphery and surfaces were obtained. These electron-donating functional groups are very important to obtain intense, stable, and excitation-independent PL emission.

**Figure 4:**
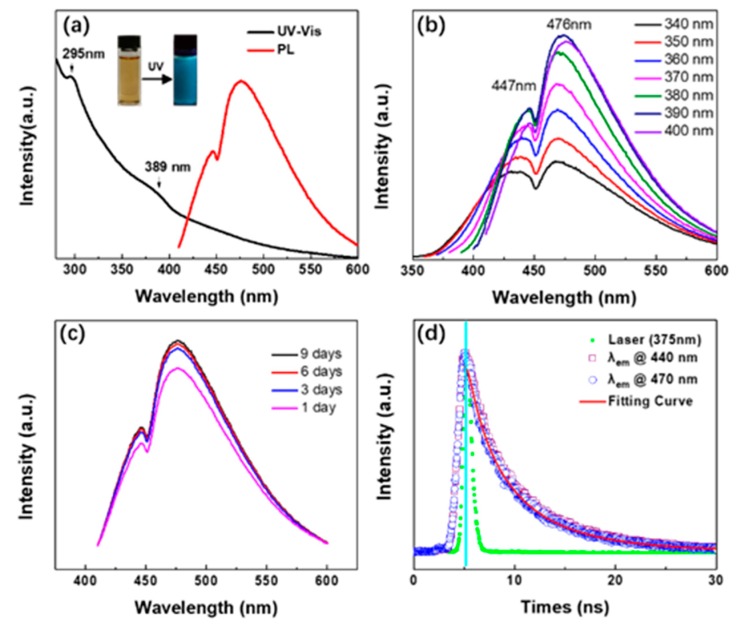
(**a**) Ultraviolet–visible light (UV–Vis) absorption and photoluminescence (PL) spectra of NM-CQDs dispersed in formamide. The insets show the photographs of NM-CQDs under a room light and a UV light source (λ = 365 nm). (**b**) PL spectra of NM-CQDs excited with different excitation wavelengths. (**c**) PL emission of NM-CQDs excited at 390 nm measured from the first to the ninth day. (**d**) PL decay profiles of NM-CQDs with two emission wavelengths of 447 nm (violet squares) and 476 nm (blue circles).

Evidently, the supernatant of NM-CQDs was yellow in color under room-light irradiation, while indigo–blue emission could be observed under UV-lamp (λ = 365 nm) irradiation in the inset of Figure 4a. Two absorption bands centered at 295 nm and 389 nm can be clearly observed in the UV–Vis spectrum. Interestingly, two coexisting distinct emission bands centered at 447 nm and 476 nm could also be seen in the PL emission spectrum. The PL emission peak centered at 476 nm was much stronger than that of the peak centered at 447 nm, displaying the indigo–blue emission. Unlike most of the reported CQDs [42,43,44], PL emission bands of NM-CQDs synthesized in formamide by PLA remained unshifted with the excitation wavelengths changing. The centers of the two bands were always situated at 447 nm and 476 nm in Figure 4b, whereas the PL intensities of the NM-CQDs increased first and then decreased as the excitation wavelengths changed from 340 to 400 nm. The PL intensity was maximum when NM-CQDs were excited with a wavelength of 390 nm. It can be noted that the intense and stable PL emissions were not derived from formamide molecules. This phenomenon can be certified by comparing the PL emission intensities of formamide (dotted line) to those of NM-CQDs (solid line) in Figure S7 (Supplementary Materials). Figure 4c shows the PL emissions of NM-CQDs with an excitation wavelength of 390 nm from the first to the ninth day. The PL emission intensities of the NM-CQDs were enhanced as time was prolonged because the agglomeration of particles made the non-radiative transitions of excited electrons reduce with prolonged time [45,46]. The intensities tended to be stable with the high quantum yield of 32.4% until the ninth day (see Figure S8, Supplementary Materials). According to previous reports [33,47,48,49], species, such as amine moieties, are crucial to improving PL properties of CQDs. Here, apart from formamide, ethyl acetate (EA) and ethylene glycol (EG) were also employed to investigate whether passivation with different solvents would impact the quantum yields of the CQDs by means of PLA. Obviously, TEM and HR-TEM images showed that NM-CQDs synthesized in different solvents were still monodispersed and possessed a distinct lattice and a micropore (see Figure S9, Supplementary Materials). Unlike NM-CQDs synthesized in formamide, non-doped CQDs, i.e., M-CQDs-EA and M-CQDs-EG, lacked N elements (see Figure S11a,b, Supplementary Materials). Compared with M-CQDs-EG, M-CQDs-EA had more C=O groups (see Figure S10c,d, Supplementary Materials). Furthermore, the quantum yields of M-CQDs-EA and M-CQDs-EG were only 15.5% and 6.3%, respectively, indicating that the nitrogen-containing groups were beneficial for increasing the QY of CQDs. As a comparation, N-CQDs from non-microporous carbon precursors were also synthesized in formamide solvent to investigate the effect of microporous structure on PL emission. TEM and HR-TEM images showed N-CQDs were absent of a microporous structure (see Figure S11a,b, Supplementary Materials). Compared with NM-CQDs, N-CQDs had fewer defects (*I_D_/I_G_* = 0.87) and showed a relatively weaker PL emission (see Figure S11c,d, Supplementary Materials), which indicated that the introduction of microporous structure can further improve the quantum yield of CQDs. Furthermore, fluorescence intensities of NM-CQDs were stable under high salt ionic concentrations (up to 400 mM NaCl) and a wide range of pH values (pH 4–9) (as shown in Figure S12a,b, Supplementary Materials). PL intensities of NM-CQDs were almost unchanged with the same excitation wavelength after 1800 s (as shown in Figure S12c, Supplementary Materials), indicating that the indigo–blue emission of NM-CQDs prepared by PLA was stable for a moment. In addition, Figure S12d (Supplementary Materials) shows that the PL intensity also decreased slightly when temperature changed from 293.15 K to 393.15 K, which indicated that the strong, stable, and excitation-independent indigo–blue PL emission of NM-CQDs originated from the surface states [43]. Finally, the PL lifetime of NM-CQDs was also detected using a fluorescence spectrometer. The differently colored discrete dots in Figure 4d display the PL decay profiles of the NM-CQDs by monitoring PL peaks at 447 nm (violet squares) and 476 nm (blue circles) with an excitation wavelength at 375 nm using a time-correlated single photon counting method. The corresponding instrument response curve is also given in Figure 4d. The lifetime data of NM-CQDs were fitted with the triplet-exponential function shown below.

(1) Iem= A1exp(−tτ1)+A2exp(−tτ2)+A3exp(−tτ3),

where *A*_1_, *A*_2_, and *A*_3_ are amplitude components of the first, second, and third decay exponents, respectively [36]. The fitted curved lines are shown as red solid lines in Figure 4d, and they are consistent with the experimental results. It was found that the decay curve contained a fast component (τ_1_) and two slow components (τ_2_ and τ_3_). The average decay time of the PL peak at 447 nm was 5.96 ns (τ_1_ = 1.793 ns, τ_2_ = 4.939 ns, τ_3_ = 10.950 ns), and that of the PL peak at 476 nm was 6.56 ns (τ_1_ = 2.138 ns, τ_2_ = 5.447 ns, τ_3_ = 12.572 ns), determined using an iterative reconvolution method (as shown in Table S1, Supplementary Materials), which are higher than those of CQDs prepared by microwave and hydrothermal methods (as shown in Table 1). Therefore, the observed lifetimes of NM-CQDs over a nanosecond suggest that the as-synthesized sample can be a desirable candidate for biological applications [36].

**Figure 5:**
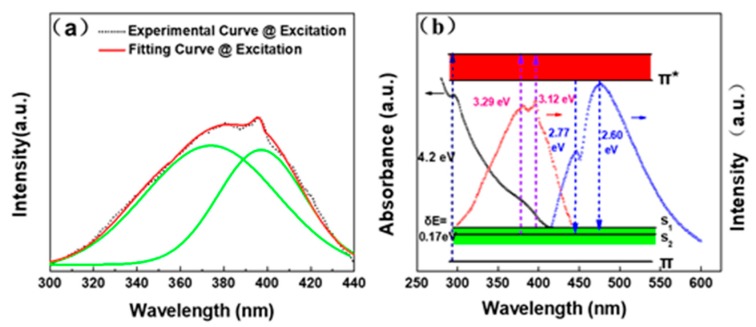
Proposed PL mechanism of NM-CQDs synthesized by pulsed laser ablation (PLA). (**a**) The measured PLE spectrum of NM-CQDs determined by monitoring the PL peak at 470 nm (dashed line), the sum of Lorentzian fittings (red line), and two Lorentzian functions (green lines). (**b**) PL emission mechanism profile of NM-CQDs.

In previous studies, some impurities were possibly produced using the acid treatment method or hydrothermal method in colloidal CQDs due to severe conditions (e.g., strong acid). Hence, the PL mechanism of CQDs remained ambiguous and is still an open issue to be discussed by researchers. In general, the PL emission of CQDs is probably derived from electron transitions of the intrinsic state [1,35,36] or the surface/edge state [54,55,56]. To clearly clarify the PL mechanism of the NM-CQDs, the photoluminescence excitation (PLE) spectrum was measured by monitoring the PL emission band centered at 470 nm; then, it was fitted into two primary components (green curves) located at 377 nm (~3.29 eV) and 397 nm (~3.12 eV) by a Lorentzian function, as shown in Figure 5a. The PLE spectrum including the dual-excitation band provides imperative theoretical evidence for further proposing a reasonable PL mechanism of NM-CQDs. In the PLA process, the local high temperature pyrolyzes the bulk PC precursor into *sp*^2^ nanoclusters (π and π* orbitals) which have many dangling bonds on the periphery, surface, and pored positions. Meanwhile, formamide solvent molecules are simultaneously pyrolyzed into nitrogen-containing and oxygen-containing functional groups which can directly passivate the edges and surfaces of PC nanoclusters to form passivation layer structures. Therefore, surface molecular orbitals (surface state) from the cracked formamide are generated between the π and π* states. In general, the surface state has a strong ability to capture electrons. The excited electrons will recombine with holes on the surface state to generate the intense and stable PL emission after vibrational relaxation. Thus, the broad absorption band centered at 389 nm is probably derived from the electron contribution of the surface state in the UV–Vis absorption spectrum. By further calculating PLE and PL spectra, the energy difference of fitting excitation peaks centered at 377 nm (~3.29 eV) and 397 nm (~3.12 eV) was approximately 0.17 eV, which was nearly equal to that of the PL emission peaks centered at 447 nm (~2.77 eV) and 476 nm (~2.60 eV) in Figure 5b. Thus, the intense and double coexisting PL emissions were attributed to electron transitions from the π* orbital to the surface molecular orbitals (S_1_ and S_2_), which were possibly related to nitrogen-containing or oxygen-containing functional groups. These functional groups are also called fluorescent chromophores, which can donate their electrons to the nanoscale microporous C domains for increasing the charge density of the core. Hence, the energy levels of NM-CQDs rose and the band gap narrowed, similar to the case of increasing the size of the CQDs to reduce the band gap. With the number of functional groups increasing, the passivation could be saturated. The band gap of NM-CQDs was invariant and insensitive to the size owing to the saturated passivation. The electron-donating functional groups played a leading role in controlling the PL emission of the NM-CQDs in wavelength and intensity. Eventually, two coexisting, intense, stable, and excitation-independent indigo–blue PL emissions of NM-CQDs could be clearly observed.

**Figure 6:**
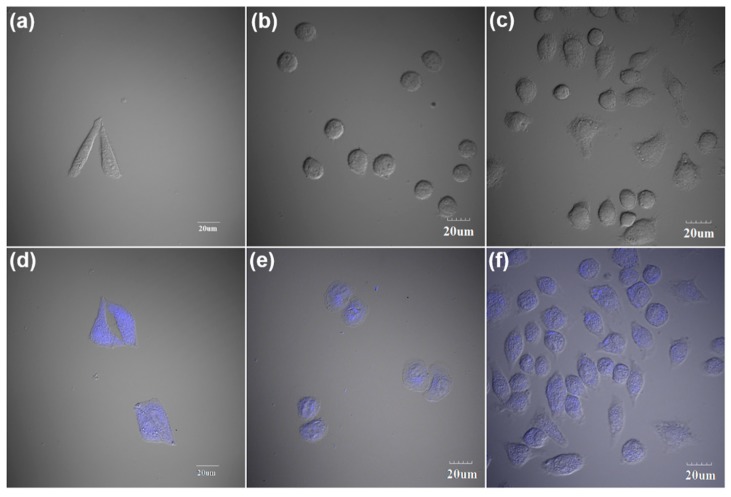
(**a**–**c**) Confocal microphotographs of live HeLa cells, L02 cells, and macrophage cells without NM-CQD incubation, respectively. (**d**–**f**) Confocal micro-photographs of live HeLa cells, L02 cells, and macrophage cells incubated with 40 µL of NM-CQDs at 37 °C for 3 h, respectively. Detection of NM-CQD fluorescence in cells was achieved using a 405-nm laser as an excitation source.

As hypothesized, 40 µL of NM-CQDs was introduced into 96-well plates holding cancer cells to incubate the HeLa cells at 37 °C for 3 h. Compared with live cells without the treatment of NM-CQDs (see Figure 6a), HeLa cells incubated with NM-CQDs showed obvious PL emissions with a 405-nm laser as an excitation source (see Figure 6d). In addition, the staining images of L02 cells (see Figure 6b,e) and macrophage cells (see Figure 6c,f) are also given, which further indicated that the formamide passivated CQDs had good internalization. Therefore, the NM-CQDs fabricated by pulsed laser ablation of the activated biomass target have great potential to be applied in biomedical imaging and engineering [57,58].

## 4. Conclusions

In this work, N-doped micropore carbon quantum dots with a high quantum yield and dual-wavelength PL emissions were fabricated using pulsed laser ablation (PLA) from low-cost, sustainable, and waste *Platanus* biomass. Two strong and coexisting PL emissions in the indigo–blue wavelength region were clearly observed, elucidating that the excited electrons transited from the intrinsic π* orbital to the surface state (SS) formed from the saturation passivation. The QY and FL of the obtained NM-CQDs were as high as 32.4% and 6.56 ns, which exceeded the non-doped CDs and those passivated by other solvents, indicating that the nitrogen-containing functional group was beneficial to improving the quantum yield. Further investigations showed that the emission behaviors of formamide passivated CQDs were still stable and independent in various conditions, such as various excitation wavelengths, salt ionic concentrations, pH values, irradiation times, and temperatures. In addition, the obtained NM-CQDs are very suitable for cellular staining images due to strong and stable PL emission in various conditions and they show good internalization in different cells. Therefore, we propose a new preparation strategy for highly dual-wavelength fluorescent NM-CQDs with great potential in biomedical imaging and engineering.

## Figures and Tables

**Table 1:** Quantum yield and lifetime of N-doped micropore carbon quantum dots (NM-CQDs) compared to other CQDs or graphene quantum dots (GQDs).

Sample	Method	Quantum Yield (%)	Lifetime (ns)	Reference
**NM-CQDs**	Pulsed laser ablation	32.4	6.56	This work
**GQDs**	Pulsed laser ablation	12	~	[30]
**N-CDs**	Hydrothermal	16	~	[34]
**GQDs**	Acid treatment	~	2.35	[36]
**GQDs**	Microwave-assisted hydrothermal	11	<6.29	[43]
**N-GQDs**	Hydrothermal	34.5	4.84	[49]
**GQDs**	Electrochemical	5.1	~	[50]
**C-nanodots**	Microwave-assisted pyrolysis	30.2	~	[51]
**CQDs**	Microwave	10	5.7 ± 0.3	[52]
**Boronic acid GQDs**	Hydrothermal and acid treatment	49.7	~	[53]

## Supplementary Materials

The following are available online at https://www.mdpi.com/2079-4991/9/4/495/s1: Figure S1: Homemade pulsed laser ablation system; Figure S2: N_2_ sorption isotherm measurement and pore size distribution of porous carbon; Figure S3: Statistical size distribution graph and DLS spectrum of NM-CQDs; Figure S4:AFM image and statistical height graph of NM-CQDs; Figure S5: XPS spectrum and high-resolution N_1S_ spectrum; Figure S6: Zeta potential measurements; Figure S7: PL spectra of NM-CQDs and formamide solvent; Figure S8: Statistical profiles of PL emission intensities of NM-CQDs; Figure S9: TEM and HR-TEM images of M-CQDs-EA and M-CQDs-EG; Figure S10: XPS spectra and high-resolution C_1s_ spectra of M-CQDs-EA and M-CQDs-EG; Figure S11: TEM images, Raman spectrum, and PL spectrum of N-CQDs from non-microporous carbon precursor treatment; Figure S12: Stability measurements of NM-CQDs under different conditions (NaCl concentrations, pH value, light irradiation time, temperature).
