# Supplementary material for: Synthesis of N-Doped Micropore Carbon Quantum Dots with High Quantum Yield and Dual-Wavelength Photoluminescence Emission from Biomass for Cellular Imaging
Source: Nanomaterials (Basel). 2019 Apr 1;9(4):495. doi: 10.3390/nano9040495 (PMC6523831; doi:10.3390/nano9040495)
Supplement: Supplementary file 1 [file nanomaterials-09-00495-s001.pdf]

Supplementary Information to

Synthesis of N-doped micropore carbon quantum dots with high quantum yield and dual-wavelength photoluminescence emission from biomass for cellular imaging

Xin Ren[1], Fang Zhang[2], Bingpeng Guo[1], Na Gao[1], and Xiaoling Zhang [1,*]

[1] *School of Chemistry and Chemical Engineering, Beijing Institute of Technology Beijing, 100081, P. R. China*

[2] *Analytical and Testing Center, Beijing Institute of Technology Beijing, 100081, P. R. China*

E-mail: zhangxl@bit.edu.cn

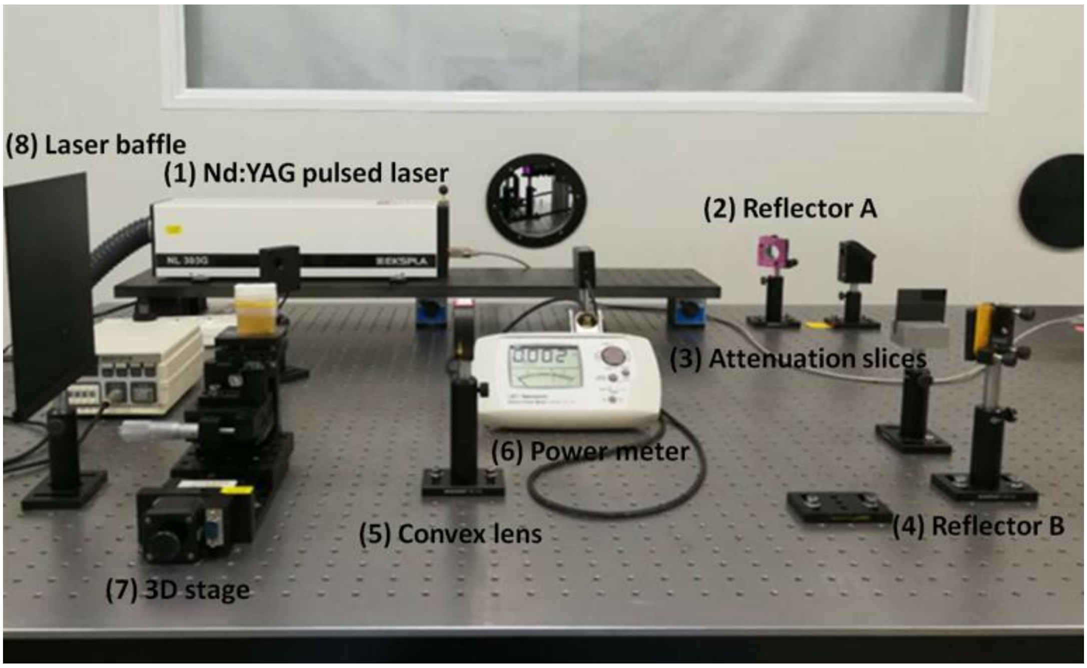

Figure S1. Home-made pulsed laser ablation system for synthesis of NM-CQDs from waste platanus biomass.

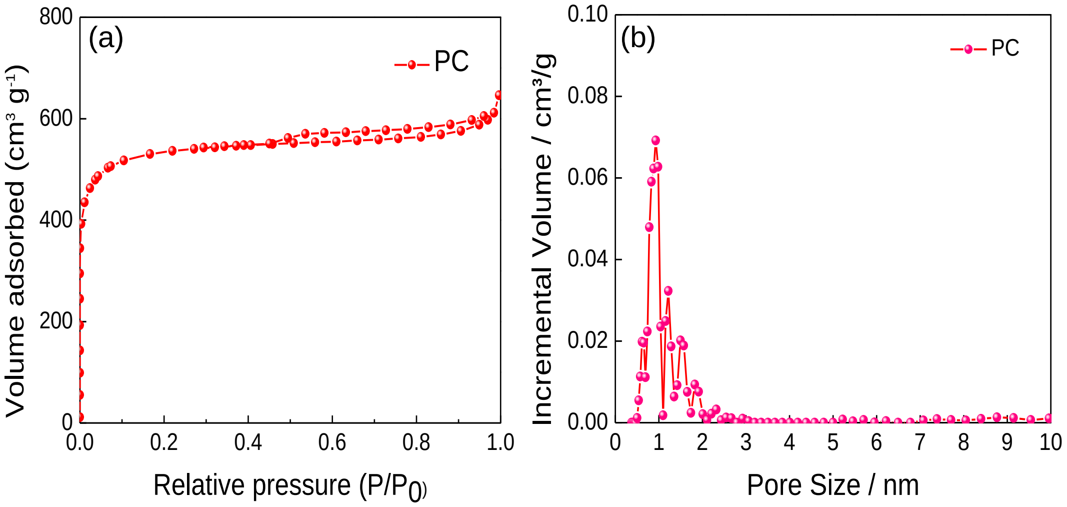

Figure S2. (a) N_2 sorption isotherm characteristic, and (b) pore size distribution of porous carbon.

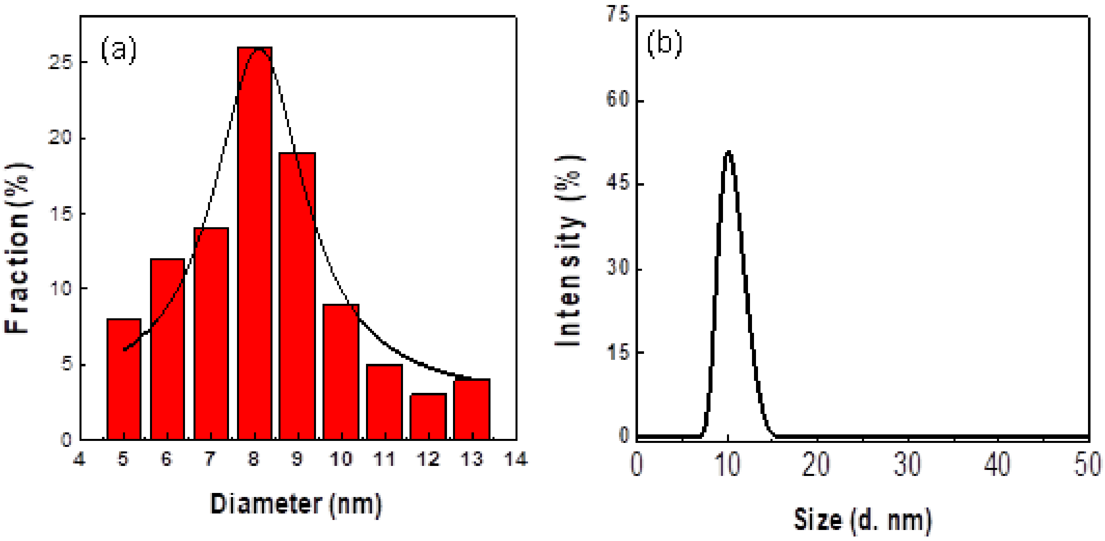

Figure S3. (a) Size distribution graph of NM-CQDs. (b) DLS spectrum of NM-CQDs.

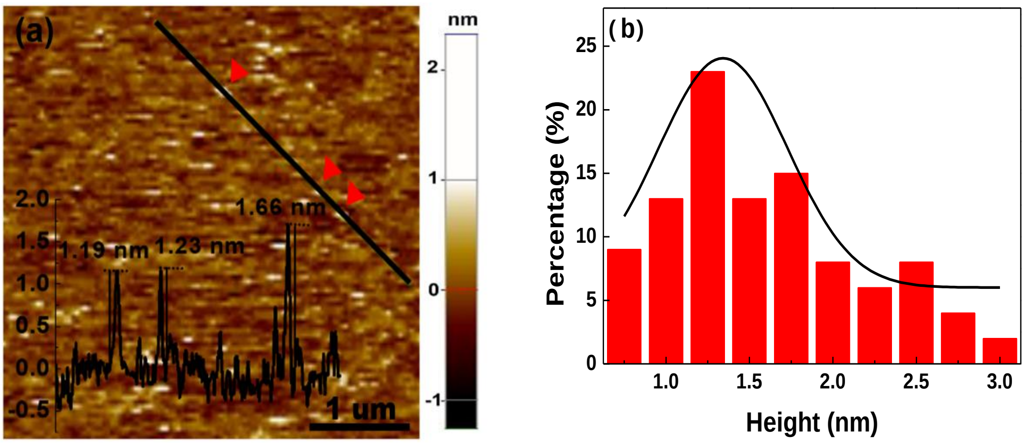

Figure S4. AFM image of NM-CQDs. Inset: distribution profiles of NM-CQDs along black line marked in Figure S5 (a). (b) Statistical height graph of NM-CQDs.

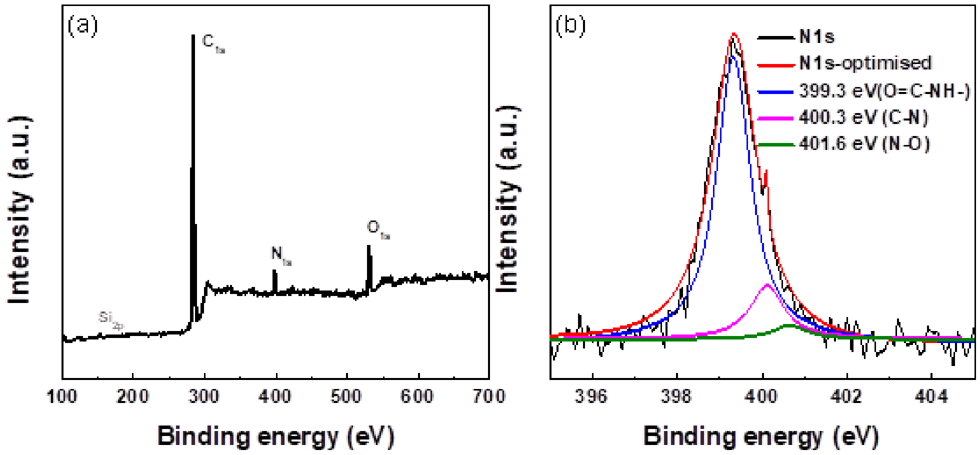

Figure S5. (a) XPS spectrum. (b) High-resolution N1S spectrum of NM-CQDs.

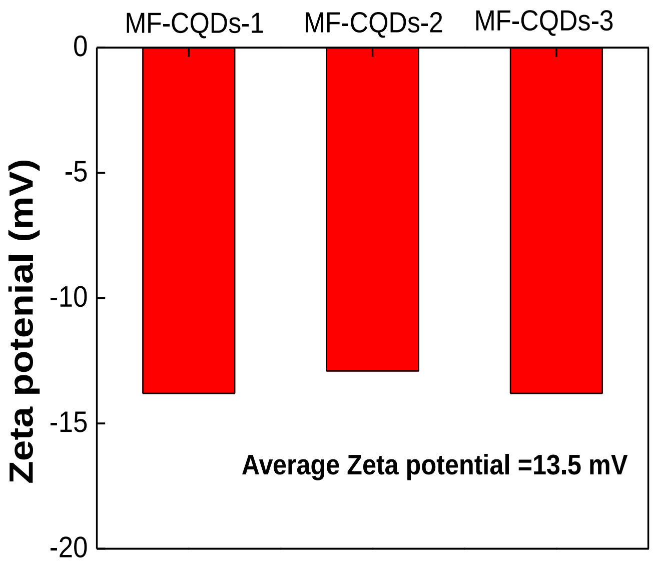

Figure S6. Zeta potential values of NM-CQDs.

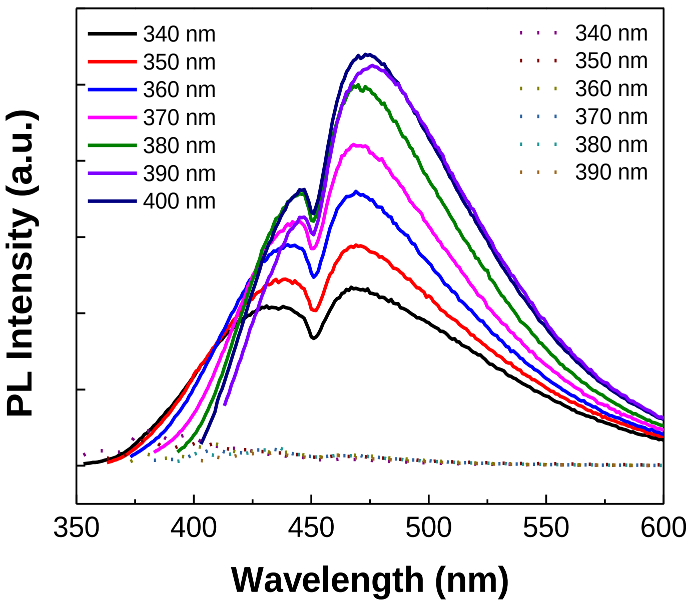

Figure S7. PL spectra of NM-CQDs (solid line) and formamide solvent (dashed line) at different excitation wavelengths ranging from 340 nm to 400 nm.

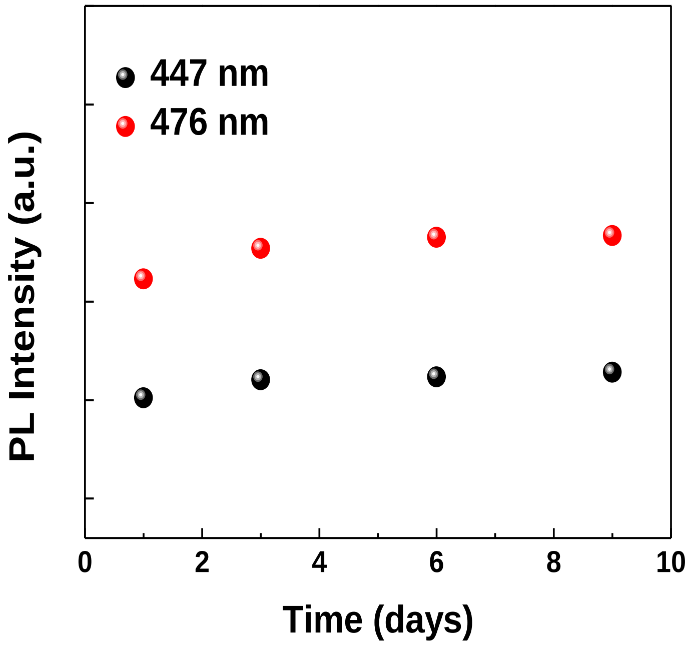

Figure S8. Statistical graph of PL emission intensities of NM-CQDs at 447 nm and 476 nm from the 1st to 9th day.

Table. S1 TCSPC data for the NMF C-QDs

**PL emission**	τ_1 (ns)	A_1	τ_2 (ns)	A_2	τ_3 (ns)	A_3	τ_{av} (ns)
**447 nm**	1.793	17.96	4.939	55.30	10.950	26.73	5.96
**476 nm**	2.138	20.42	5.447	54.50	12.572	25.08	6.56

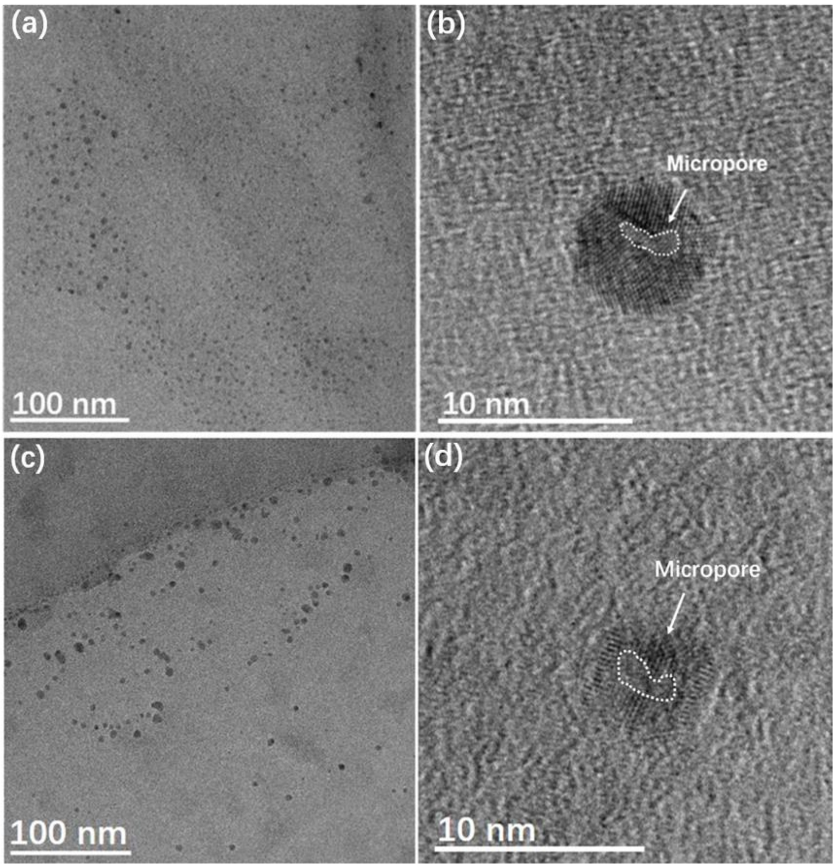

Figure S9. TEM and HR-TEM images of M-CQDs passivated by substituents from different solvents by PLA. (a)-(b) Ethyl acetate, (c)-(d) Ethylene glycol.

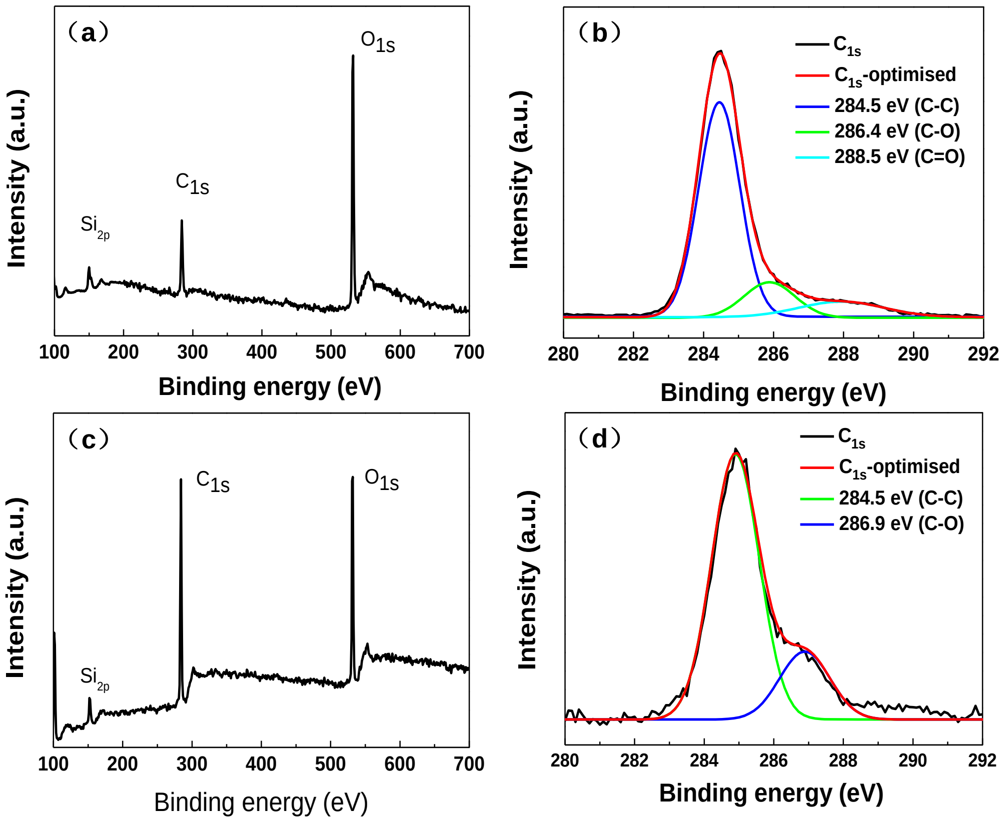

Figure S10. XPS spectrum and High-resolution C_{1s} spectrum of M-CQDs passivated by substituents from different solvents by laser ablation. (a) Ethyl acetate, (b) Ethylene glycol.

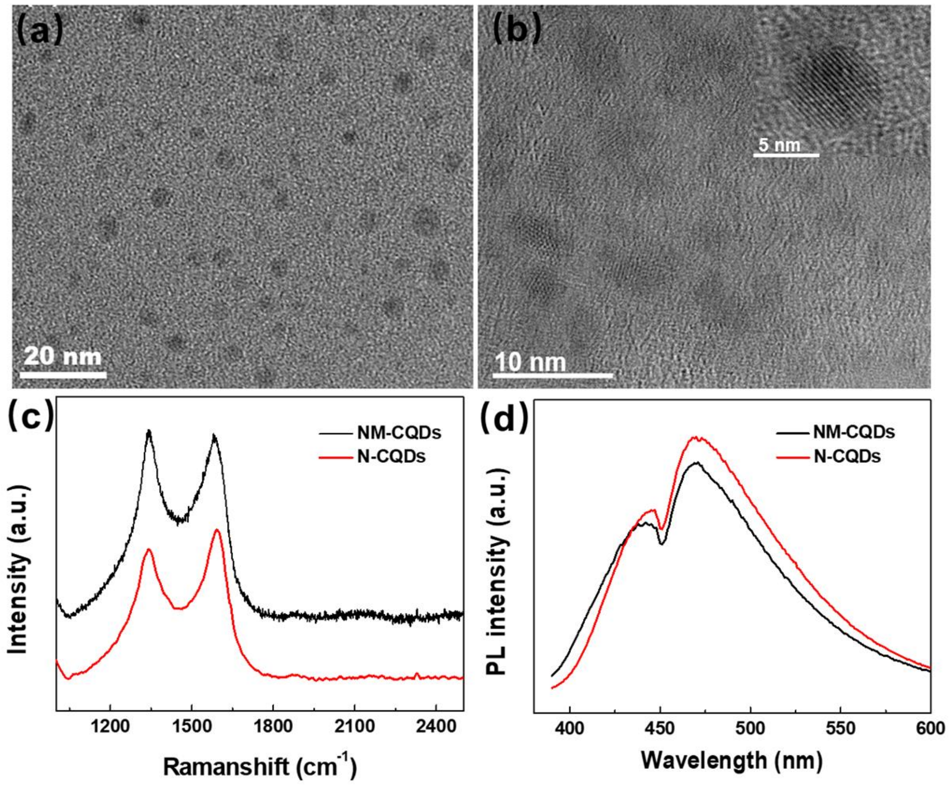

Figure S11. (a)-(b) TEM and magnifying TEM images of N-CQDs from non-microporous carbon precursor treatment without KOH. The inset is a HR-TEM image of CQDs (c) Raman spectra of NM-CQDs and N-CQDs with an excitation wavelength of 532 nm. (d) PL emission of NM-CQDs and N-CQDs with an excitation wavelength of 380 nm.

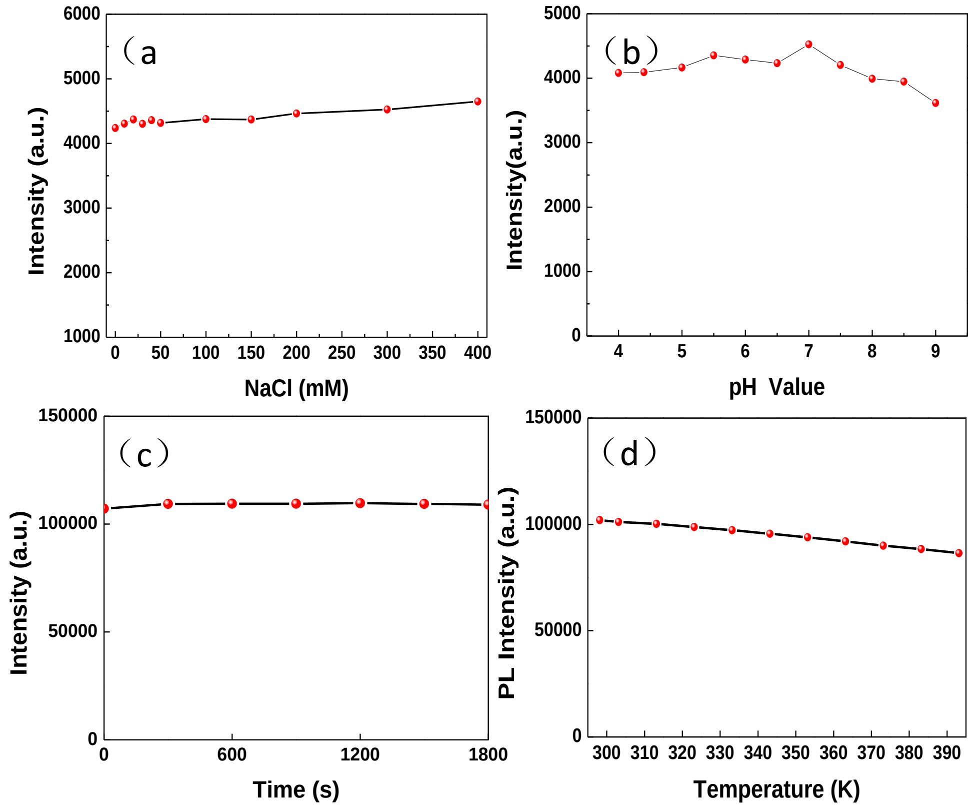

Figure S12. (a) PL intensities of NM-CQDs after adding various concentrations of NaCl (0, 10, 20, 30, 40, 50, 100, 150, 200, 300, 400 mM) in PBS (pH 7.4). (b) PL intensities of NM-CQDs at different pH values. (c) PL intensities of NM-CQDs with an excitation wavelength at 390 nm after 1800 seconds. (d) PL intensities of NM-CQDs at different temperatures from 293.15K to 393.15K.
